# Enhanced bone tissue regeneration using a 3D-printed poly(lactic acid)/Ti6Al4V composite scaffold with plasma treatment modification

**DOI:** 10.1038/s41598-023-30300-z

**Published:** 2023-02-23

**Authors:** Masoud Zarei, Motahareh Shabani Dargah, Mahdi Hasanzadeh Azar, Reza Alizadeh, Fatemeh Sadat Mahdavi, Sayed Shahab Sayedain, Alireza Kaviani, Mohammad Asadollahi, Mahmoud Azami, Nima Beheshtizadeh

**Affiliations:** 1 Department of Materials Science and Engineering, Sharif University of Technology, Tehran, Iran; 2 Department of Biomedical Engineering, Amirkabir University of Technology, Tehran, Iran; 3 Department of Engineering Physics, McMaster University, Hamilton, Canada; 4 Department of Biotechnology Engineering, College of Science, University of Tehran, Tehran, Iran; 5 Polymeric Materials Research Group (PMRG), Department of Materials Science and Engineering, Sharif University of Technology, Tehran, Iran; 6 Department of Tissue Engineering, School of Advanced Technologies in Medicine, Tehran University of Medical Sciences, Tehran, Iran; 7 Regenerative Medicine Group (REMED), Universal Scientific Education and Research Network (USERN), Tehran, Iran; 8 Joint Reconstruction Research Center (JRRC), Tehran University of Medical Sciences, Tehran, Iran

**Keywords:** Biomedical engineering, Tissues, Mechanical properties

## Abstract

The mechanical and biological properties of polylactic acid (PLA) need to be further improved in order to be used for bone tissue engineering (BTE). Utilizing a material extrusion technique, three-dimensional (3D) PLA-Ti6Al4V (Ti64) scaffolds with open pores and interconnected channels were successfully fabricated. In spite of the fact that the glass transition temperature of PLA increased with the addition of Ti64, the melting and crystallization temperatures as well as the thermal stability of filaments decreased slightly. However, the addition of 3–6 wt% Ti64 enhanced the mechanical properties of PLA, increasing the ultimate compressive strength and compressive modulus of PLA-3Ti64 to 49.9 MPa and 1.9 GPa, respectively. Additionally, the flowability evaluations revealed that all composite filaments met the print requirements. During the plasma treatment of scaffolds, not only was the root-mean-square (*R*q) of PLA (1.8 nm) increased to 60 nm, but also its contact angle (90.4°) significantly decreased to (46.9°). FTIR analysis confirmed the higher hydrophilicity as oxygen-containing groups became more intense. By virtue of the outstanding role of plasma treatment as well as Ti64 addition, a marked improvement was observed in Wharton's jelly mesenchymal stem cell attachment, proliferation (4′,6-diamidino-2-phenylindole staining), and differentiation (Alkaline phosphatase and Alizarin Red S staining). Based on these results, it appears that the fabricated scaffolds have potential applications in BTE.

## Introduction

Humans' bones have been prone to severe illnesses and defects such as trauma, osteoporosis, and cancer^1^. To heal these diseases, it has been commonplace to implant non-biodegradable metallic substances, generally based on titanium and stainless steel, in the body^2^. However, this approach provoked secondary surgeries to extract the metal from the body, squandering time and materials^3^. The use of bone substitutes has recently attracted the attention of many experts for repairing damaged bones without facing disasters associated with conventional methods^4^. This is mainly because these biodegradable materials are preferable sites for various types of cells to grow and regenerate new bone tissue^5^. However, it is crucial to mention that the kind of substitute plays the most influential role, as it is required to show similar features as the initial bone. These vital characteristics include proper mechanical stability, ensuring adherence, proliferation, and differentiation of cells responsible for bone regeneration^6^. Most fabricated bone substitutes have been based on biocompatible metals and ceramics, which indicate sufficient strength^7^. However, some drawbacks limit the usage of these substances in bio-based applications. One of the main problems is that the modulus of most ceramics and metals is significantly higher than that of bones, which makes the load transfer unbalanced and causes bone resorption as a consequence of the stress shielding effect^8,9^. In addition, several defects in ceramics diminish their ability to demonstrate acceptable toughness. Also, around metallic implants, some artifacts appear during magnetic resonance imaging (MRI) and computerized tomography (CT) scanning, invalidating the accuracy of results^10^. Therefore, finding a promising biomaterial candidate has remained a great challenge for researchers.

Currently, biocompatible polymers, like polyetheretherketone (PEEK), polycaprolactone (PCL), and polylactic acid (PLA), show high potential for use in bone tissue engineering (BTE), thanks to their biodegradability, non-toxicity, non-immunogenic, and non-inflammatory properties ^11^. Among a wide range of polymeric compounds, PLA, a thermoplastic aliphatic polyester, has been introduced as one of the best candidates in hard tissue engineering. This cost-effective polymer possesses low melting temperature and acceptable biocompatibility, paving the way for utilization in BTE^12^. The advantages of PLA notwithstanding, the low biological activity and mechanical properties are considered demerits that should be addressed^13^.

A number of attempts have been made to improve the performance of PLA scaffolds by adding external reinforcements. PCL, for example, has proven to be one of the most widely used polymers due to its biocompatibility and comparability with the rate of bone regeneration. However, PLA/PCL composites may also have inferior mechanical properties, such as a low modulus or strength^14^. In another study, when *β*-tricalcium phosphate (β-TCP) was embedded in the PLA as a stiff ceramic filler, the toughness and ductility were decreased, which was attributed to the ununiformed particle distribution^15^. While ceramics and polymers were incapable of improving mechanical properties of PLA, metallic fillers were proposed as effective alternatives due to their fascinating biocompatible and toughness features. For instance, One of the recent studies indicated that the compressive strength of PLA matrix was enhanced by 8.3% by incorporating 5 wt% of magnesium powders^16^.

Various manufacturing methods have been employed in the last decade to fabricate bio-composite scaffolds, including molding, gas foaming, solvent casting, extrusion, polymerization, and electrospinning^17,18^. However, in most cases, these approaches failed to show acceptable mechanical and biological performance due to the negative impact of solvent or residual particles toxicity^19^. In recent years, 3D printing has been introduced as an efficient method to prepare complex porous scaffolds for tissue engineering by controlling the internal geometry and pore structures^20,21^. Among different types of 3D-printing approaches, polymeric samples are mainly prepared by fused deposition modeling (FDM) and selective laser sintering (SLS) techniques^22,23^. FDM offers greater flexibility in material handling and better cost-effectiveness in producing porous structures without employing solvents as with stereolithography (SLA) and SLS^24,25^. This eye-catching process involves extruding a thermoplastic-based feeding filament from a heated nozzle along with solidifying the filament beads on the printing bed, which is desirable for fabricating composite scaffolds^26^.

Although some studies have been conducted to fabricate and examine polymer/metal composites printed scaffolds and have yielded promising results, some issues are still remained. As an example, 3D-printed PLA/Titanium^27^ and PLA/pure iron^28^ scaffolds were manufactured using FDM technique; however, even though these studies achieved useful results, they did not demonstrate that high-quality filament (with consistent diameter, sufficient particle dispersion, and negligible porosity) can be fabricated using this technology. In another investigation^29^, 3D-printed PLA/Zinc scaffolds were printed using FDM technology; however, no biological analysis was carried out and the scaffolds failed to show any improvement in mechanical properties. Furthermore, Cifuentes et al.^16^ and Jiang et al.^30^ produced 3D-printed PLLA/Magnesium and PLA/316L stainless steel scaffolds, respectively, but did not include any evidence regarding rheological and biological assessment.

The Ti6Al4V (Ti64) alloy, as the most widely used metallic alloy in the additive manufacturing of orthopedic implants^31^, offers a number of advantages, including excellent biocompatibility when in contact with bone and tissues, a high probability of promoting osteoblast maturation, as an osteogenic environment is established when cells similar to osteoblasts are cultured on its substrate, which could improve bone formation^32^. In spite of this, the most common additive manufacturing techniques for fabricating Ti64 implants are SLS, selective laser melting (SLM), and electron beam melting (EBM)^33^. Numerous studies have been conducted regarding the development and enhancement of Ti64 orthopedic implants as fully metallic implants so that they can meet the requirements of bone implants^34–38^. To the best of our knowledge, however, Ti64 has not yet been investigated for use as a metallic additive in material extrusion additive manufacturing (MEAM) techniques, such as FDM and SLA, all of which are used to fabricate polymer-based composite scaffolds.

Even though 3D-printed scaffolds provide lots of places for cells to attach to, their properties can be further improved with some post-treatments^39^. According to previous works, the biocompatibility of orthopedic materials, cell adhesion, proliferation, and differentiation of cells can be improved using a broad spectrum of surface modification methods, such as biofunctionalization, coating, and non-thermal plasma treatment^40,41^. As compared to the above mentioned processes, plasma treatment has been reported to produce nano-roughness on the scaffold surface, change the wettability, and modify the surface with functional groups like oxygen atoms (i.e., reactive oxygen species (ROS)) with lower fabrication costs and complexity^42^. For instance, after plasma treatment of the PCL/HA/MgO scaffold with O_2_ and N_2_, the physical surface morphology and surface chemical properties of the 3D scaffold increased the adhesion, proliferation, and differentiation of MC3T3-E1 cells in the PCL/HA/MgO scaffolds^43^.

**Figure 1:**
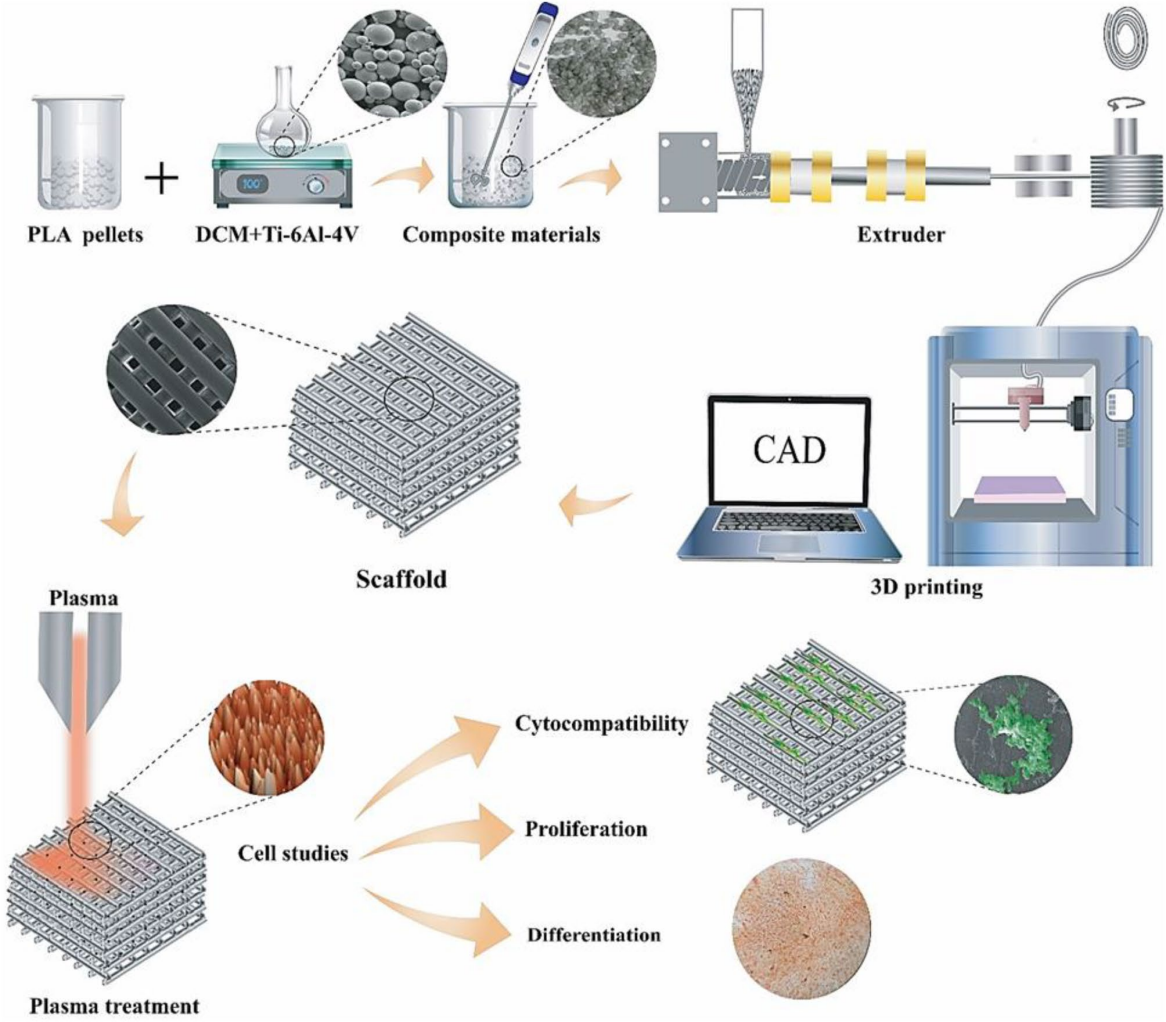
Schematic illustration of the method used to fabricate 3D porous PLA/Ti64 composite scaffolds.

Accordingly, this study was undertaken to investigate the ability of Ti6Al4V (Ti64) as an alternative PLA metallic reinforcing agent. By utilizing FDM-based 3D printing, 3D scaffolds of PLA-Ti64 with various contents of Ti64 (0, 3, 6, and 9 wt%) were constructed and investigated from the point of view of mechanical, physical, thermal, flowability, biological, and microstructural characteristics. As part of the study, an optimized concentration of Ti64 was chosen based on mechanical properties and a cell viability assay. Afterward, the selected PLA-Ti64 scaffold was treated with oxygen and air plasma to determine how plasma treatment affects scaffolds' physical and biological properties. To do so, adhesion, proliferation, and differentiation of Wharton's jelly mesenchymal stem cells were assessed on the plasma-treated scaffolds. Figure 1 depicts a schematic illustration of the 3D porous PLA/Ti64 composite scaffold preparation and characterization steps.

## Results and discussion

### Microscopy characterization of PLA/Ti64 composites

**Figure 2:**
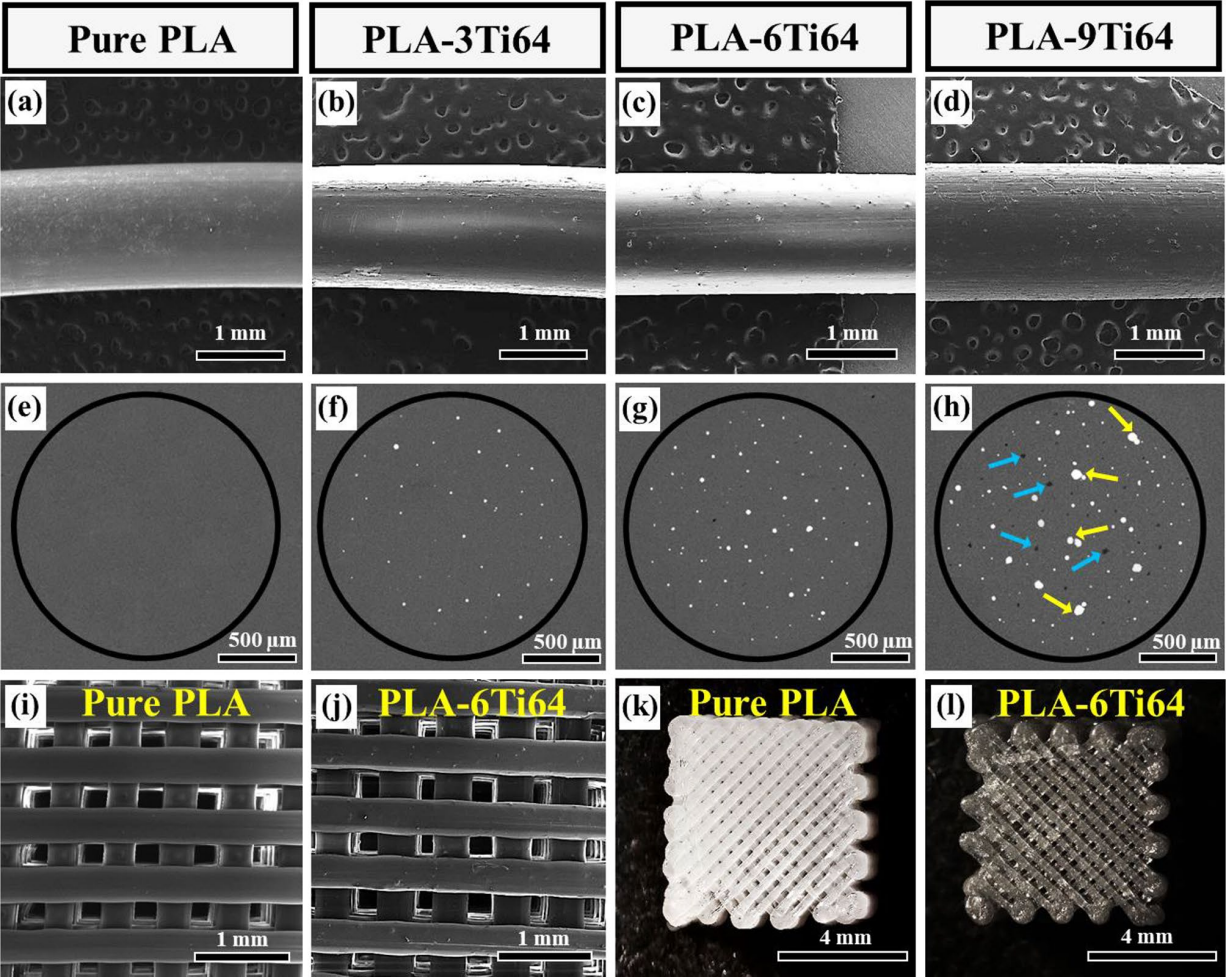
FESEM images showing the surface morphology (**a**-**d**) and cross-sectional views (**e**–**h**) of pure PLA (**a**, **e**), and composite filaments containing 3 wt% (**b**, **f**), 6 wt% (**c**, **g**), and 9 wt% (**d**, **h**) Ti64. Blue and yellow arrows in (**h**) are related to microvoids and agglomerations, respectively. FESEM with a secondary mode (**i**, **j**) and stereomicroscope (**k**, **l**) images of pure PLA (**i**, **k**) and PLA-6Ti64 (**j**, **l**) 3D-printed scaffolds.

It has been a great challenge to fabricate bio-composites in which reinforcements are uniformly distributed through the matrix. According to previous works, to overcome this problem, the optimized amount of filler should be selected^44^. Therefore, in the first step, the effect of different concentrations of Ti64 (0, 3, 6, 9 wt%) on the surface morphology of the PLA filaments was studied via field emission scanning electron microscope (FESEM). As is seen in Fig. 2a–d, when the amount of filler increased, the surface became relatively rougher, and more scratches were found. On one side, this roughness can be attributed to the Ti64 microparticles distributed on the surface of filament. On the other side, a friction force that is applied to the molten composite during the extrusion process induces the fillers to leave some tails in the form of jagged lines on the PLA surface^30^. Furthermore, based on Fig. 2e–h, which indicate cross-sectional views perpendicular to the filament axis, even though the distribution of the white Ti64 particles was found homogenous in PLA-3 wt%Ti64 (PLA-3Ti64) and PLA-6 wt%Ti64 (PLA-6Ti64) samples, some agglomerations emerged in the PLA-9 wt%Ti64 (PLA-9Ti64) filament, which corresponds to the microvoids formation in PLA^45^. These agglomerated particles played a destructive role as strong barriers against PLA flowing and hindered the matrix condensation^46^. Figure 2i–l compare the FESEM and optical images of pure PLA and PLA-6Ti64 3D-printed samples. Apparently, absence of defects such as microcracks, delamination, and voids on the struts of the scaffolds demonstrates high quality of the printed samples. In order to determine how accurate 3D-printed scaffolds were in terms of their dimensions, the size of pores and struts was measured. Based on the statistical analysis, there was no noticeable change in the pore size, porosity, and strut width of samples, recorded to be 270 µm, 35%, and 380 µm, respectively, which were slightly smaller than the adjusted values (300 µm, 40%, and 400 µm). Such slight differences arise from the volume shrinkage during the printing process due to the negative thermal gradient from the nozzle to the printing bed^47^. These open pores and interconnected channels play an outstanding role in providing the cells with more active sites to infiltrate and grow and facilitate the transport of nutrients, oxygen, and metabolic wastes^48^.

It is worth mentioning that not only are Ti, Al, V, C, and O peaks present in energy-dispersive *X*-ray spectroscopy (EDX) analysis presented in Fig. S1, which are related to Ti64 and PLA counterparts, but also the absence of chloride ions in the filaments might be a positive sign that the hazardous material has been removed completely from the composite filament during the manufacturing process.

### Thermal and flowability characterization of PLA/Ti64 composites

**Figure 3:**
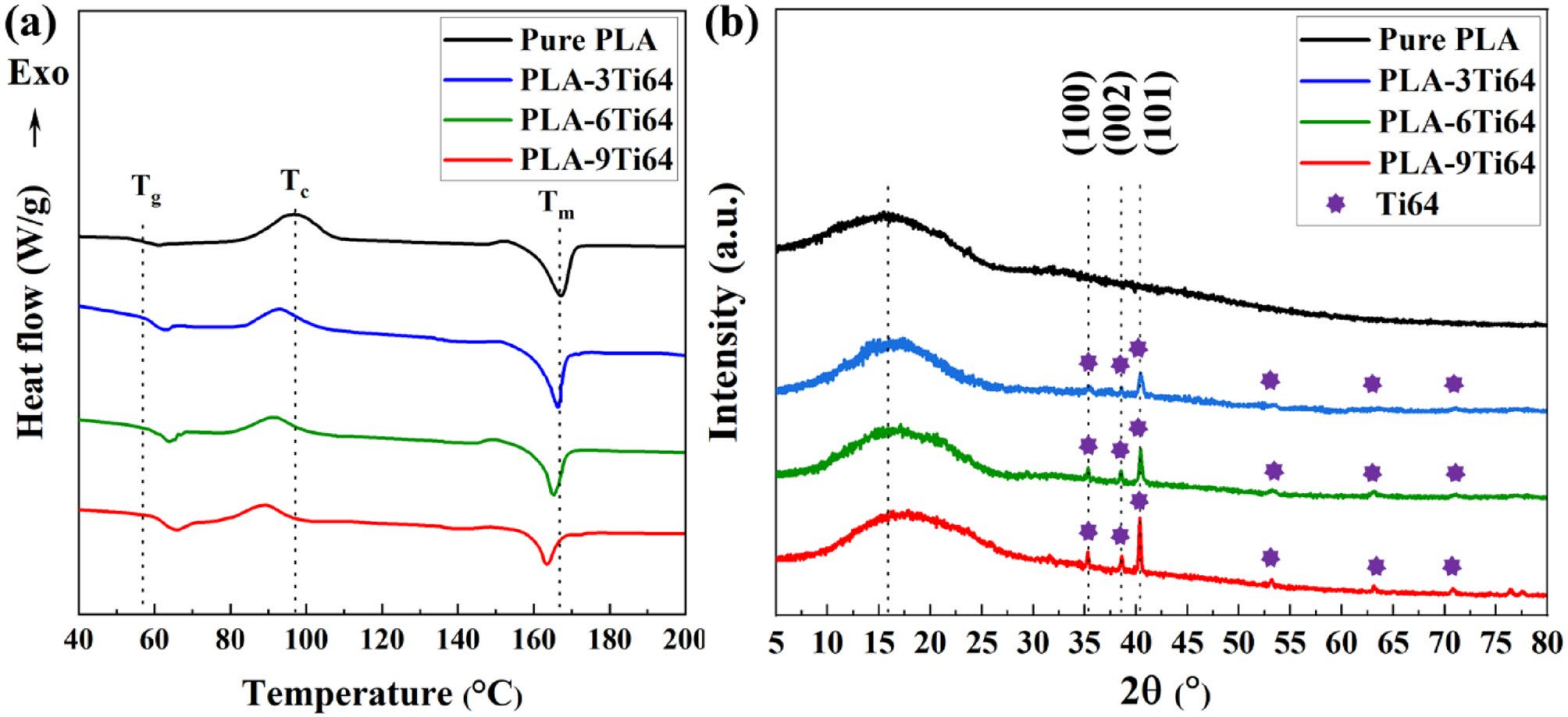
DSC curves of pure PLA filament and composite filaments with various Ti64 contents (**a**). XRD results of 3D-printed samples (**b**).

The thermal properties of PLA were evaluated by differential scanning calorimetry analysis (DSC) analysis to determine the impact of Ti64 content. The glassy temperature (*T*_g_) is an important parameter that indicates the chain mobility of polymers concerning the temperature^49^. It has been reported that the value of *T*_g_ is highly dependent on the type of reinforcement. For instance, while the hydroxyapatite/silver core–shell (HA@Ag) structure could escalate the glassy temperature of PLA, Fe_3_O_4_ particles exhibited a reverse trend with increasing filler contents^50,51^. In our survey, similar to the HA@Ag/PLA structure, when more Ti64 particles were loaded into the pure PLA matrix, the *T*_g_ of PLA (57 °C) experienced an upward trend and reached 59.8 °C, 61.6 °C, and 62.7 °C, for 3, 6, and 9 wt% Ti64 containing composites, respectively (Fig. 3a and Table 1). A slight increase in *T*_g_ can be attributed to the interfering role of Ti64 particles, which hindered the chain mobility and lubrication of PLA^52^. However, in contrast to the changes in *T*_g_, by increasing the concentration of Ti64, the cold crystallization temperature (*T*_c_) gradually declined from 97 (pure PLA) to 89.4 °C (PLA-9Ti64). The more Ti64 particles, the more nucleation sites, providing the preferable places for PLA to crystallize, resulting in a decrease in cold crystallization temperature^27^. Another critical parameter that is influenced by the concentration of Ti64 is the crystallization capacity of PLA (*X*_c_). While the value of *X*_c_ rose with the addition of Ti64 and reached the maximum figure of (8.7%) for the PLA-6Ti64 sample, the trend was reversed, and *X*_c_ fell to 4.5% for the PLA-9Ti64 filament. The first observation is probably attributed to the dispersed Ti64 particles as nucleation agents, which paved the way for the crystallization of PLA via heterogeneous nucleation. However, the fall is probably related to the agglomerated Ti64 particles considered as concentration stress centers, restricting the PLA mobility chains and crystallization^53^. In addition, by increasing the amount of Ti64 content, the PLA melting point exhibited the same declining trend as *T*_c_. Ti64 particles with the same role in crystallization can play as nucleation sites, accelerating the melting of preformed crystals at lower temperatures during heating^54^.

**Table 1:** Extracted numerical values of different parameters in DSC for PLA-Ti64 composites.

Material	*T*_g_ (°C)	*T*_c_ (°C)	*T*_m_ (°C)	*ΔH*_M_ (J/g)	*ΔH*_C_ (J/g)	*X*_c_ (%)
Pure PLA	P	97.0	167.0	26.2	24.0	2.4
PLA-3Ti64	59.8	93.0	166.3	22.4	16.1	6.9
PLA-6Ti64	61.6	91.7	165.1	19.9	11.8	8.7
PLA-9Ti64	62.7	89.4	163.6	15.1	10.9	4.5

*X*-ray diffraction (XRD) analysis was also conducted to investigate the chemical composition and phase crystallization of different 3D-printed PLA-Ti64 composites. As is illustrated in Fig. 3b, a broad peak in pure PLA, located at the diffraction angle of 16°, demonstrates the formation of the amorphous phase of the polymer^55^. As the concentration of Ti64 increased, not only did the sharp main peaks at 35°, 38°, and 40°, which are related to the {100}, {002}, and {101} planes of Ti64, become more intense, but also the PLA crystal peak became narrower and sharper^50^. It is possible to explain the first observation by the fact that the peak intensity of Ti64 leads directly to a rise in Ti64 concentration. The second one, however, comes from the crystallinity enhancement of PLA in accordance with the DSC results.

**Figure 4:**
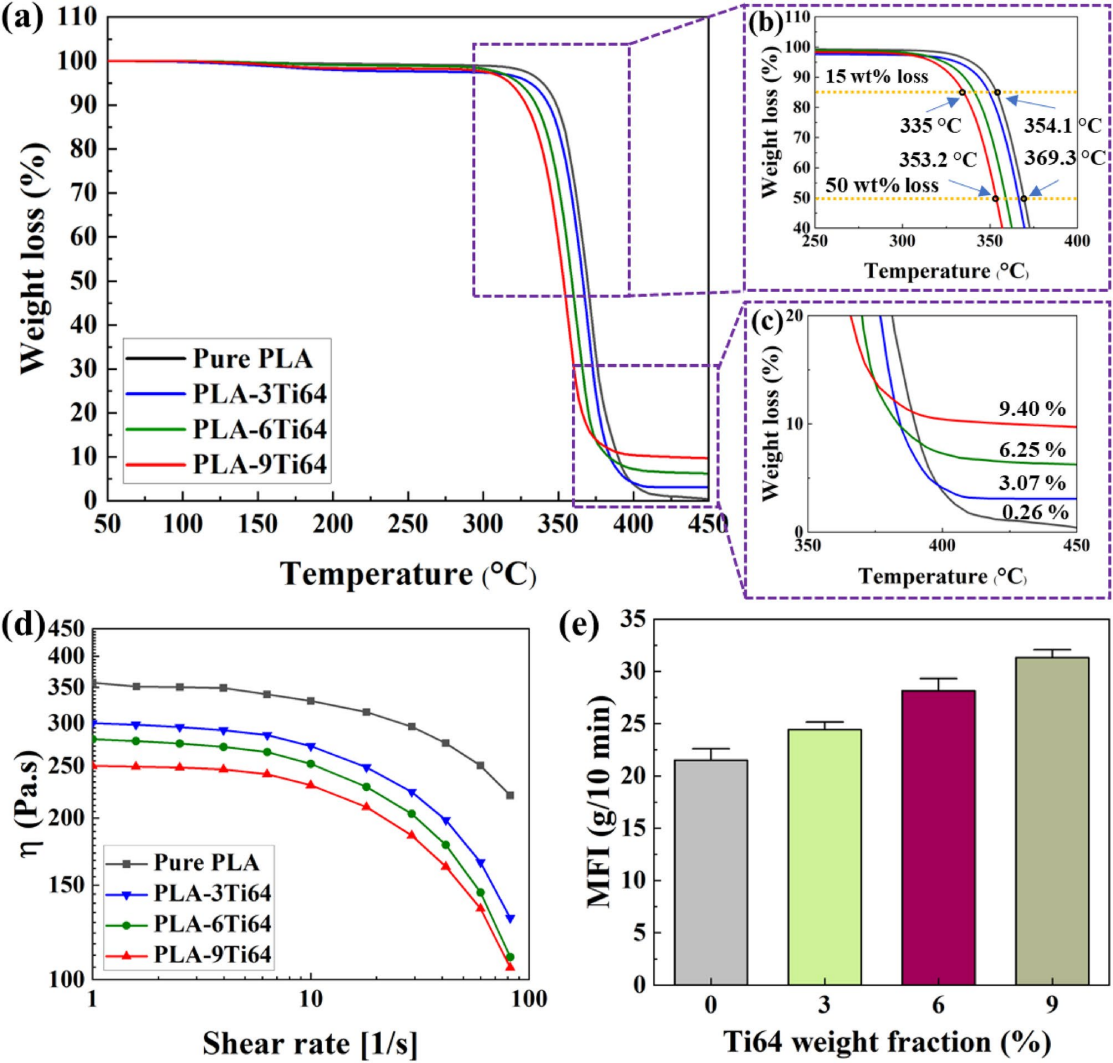
TGA (**a**–**c**), shear viscosity curves (**d**), and MFI (**e**) results obtained from PLA-Ti64 composite filaments.

Thermal stability is a vital property that manifests the printing capability of samples. It is obvious that high or low levels of temperature degradation adversely impact the quality of 3D-printed samples^56^. To end this, tuning the value of this parameter in the acceptable range above and near window temperature is crucial. To investigate the thermal stability of filaments, the changes in weight loss as a function of temperature were plotted in Fig. 4a-c. To do so, different temperatures, including onset degradation temperature (*T*_0_), the temperature of 15% weight loss (*T*_15_), the temperature of 50% weight loss (*T*_50_), and the temperature where decomposition ends (*T*_e_), are introduced and marked. First, only one thermal decomposition step was observed in the temperature interval of 300 °C to 410 °C in all the samples. When higher amounts of Ti64 were added to the pure PLA samples, all the above-mentioned temperatures decreased, showing that the particles accelerated the PLA degradation^16^. Particles of Ti64 have a large number of active sites as well as adsorbed and absorbed water on their surfaces, thereby acting as depolymerization catalysts for PLA degradation^50^. Another probable reason can be attributed to molecular weight loss and the presence of shorter chains^16^. It is noteworthy that, despite the downward shift in degradation temperatures for Ti64-loaded PLA samples, the measured values are still above the extrusion and 3D-printing temperatures (window temperatures), which are 195 and 210 °C, respectively, demonstrating the samples' excellent printing capabilities. Furthermore, the remnant mass calculated from the analysis was fairly consistent with the nominal values for composite filaments with fillers content up to 6 wt%, exhibiting Ti64 particles are uniformly dispersed within the PLA matrix. However, the difference between the obtained and calculated values for 9 wt% Ti64 is a sign of inhomogeneous distribution of particles in the PLA observed in FESEM results^27^.

Rheological and melt flow properties are also generally considered two essential factors for the assessment of the material extrusion processability and printability of polymers and polymer composites^57,58^. As shown in Fig. 4d, the flow curve of all filaments displayed a zero-viscosity range followed by an exponential decay of measured viscosity, indicating shear thinning behavior at higher shear rates. In this study, a nozzle diameter of d = 0.4 mm at a printing speed of v = 35 mm/s was used such that the approximate printing shear rate was estimated to be $\dot{\gamma }$ ≈ v⁄d ≈ 90 s^−1^ ^59^. Therefore, the remarkable non-Newtonian characteristic of filaments ensures a significant reduction in viscosity at the nozzle during the material flow at the estimated printing shear rate and, subsequently, a rapid increase in viscosity when the material is deposited on the bed. Moreover, the viscosity of neat PLA was reduced when incorporated with Ti64 microparticles. The viscosity reduction in composite filaments can be attributed to the role of micron-sized particles in lowering friction of melt and degradation of PLA molecule chains which was confirmed by thermogravimetric (TGA) analysis^50,59^. Although the viscosity values of composite filaments were found to be lower than that of neat PLA, the figures still lie in the range of 100–300 Pa.s. According to the literature, such viscosity range can guarantee the flowability of materials and retain structural integrity during and after the printing process, respectively ^59^. Figure 4e illustrates the results of melt flow index (MFI) testing for PLA and PLA-Ti64 composite filaments. Similar to the observed trend in the rheological study, the average values of MFI for composites were superior to neat PLA. In addition, the MFI slightly increased with the Ti64 content, ranging from 24 to 31 (g/10 min). These results show that MFI values fall within the printability window in the material extrusion technique^45^. The higher flowability of the PLA/Ti64 matrix brought about thin and uniform layers during 3D printing.

### Mechanical characterization of PLA/Ti64 scaffolds

**Figure 5:**
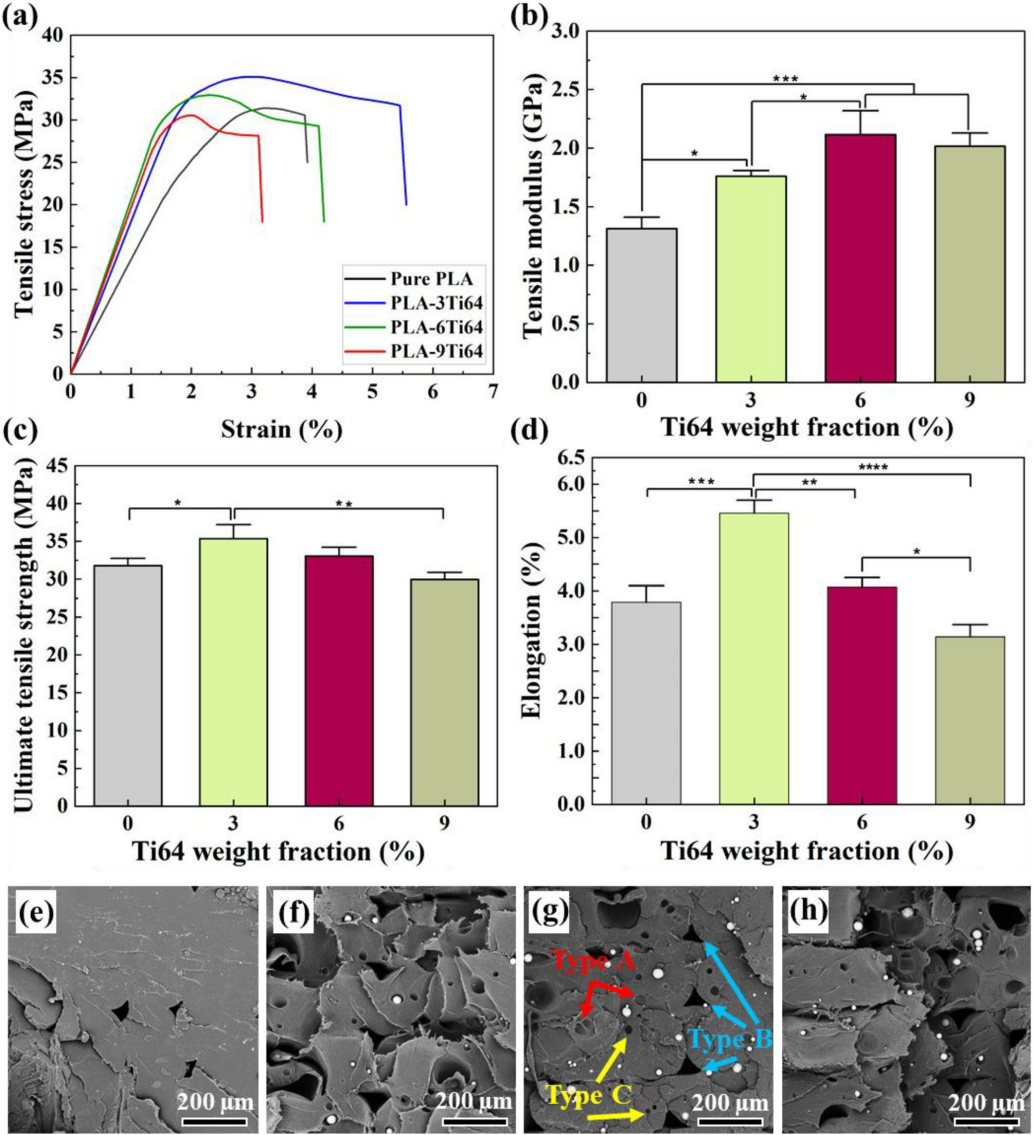
Tensile curves (**a**), tensile modulus (**b**), ultimate tensile strength (**c**), and elongation (**d**) of PLA/Ti64 composite scaffolds (*p < 0.05, **p < 0.01, ***p < 0.001, ****p < 0.0001). FESEM micrographs from fracture surfaces of pure PLA (**e**), PLA-3Ti64 (**f**), PLA-6Ti64 (**g**) and PLA-9Ti64 (**h**) scaffolds.

**Figure 6:**
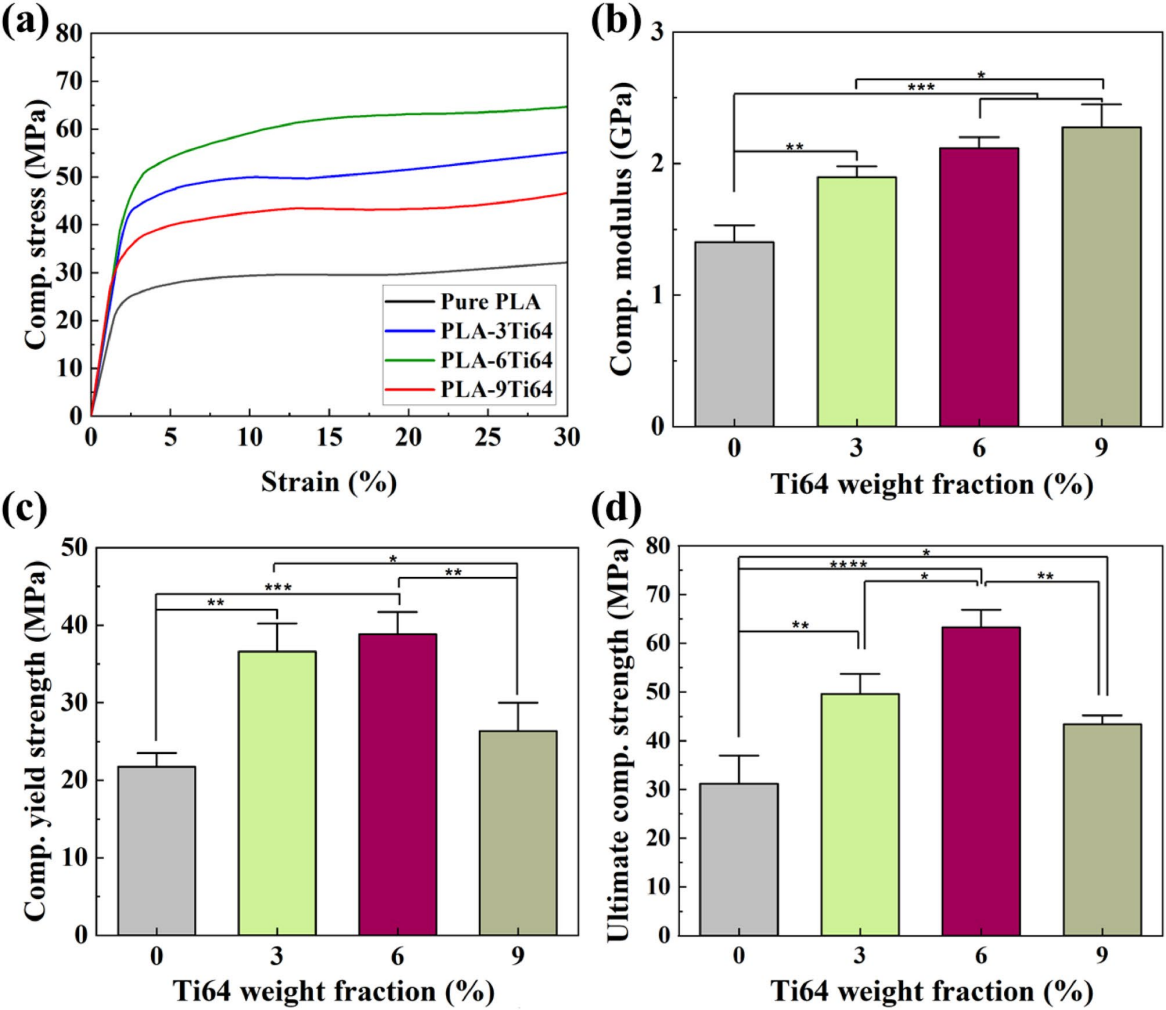
Compressive curves (**a**), compressive modulus (**b**), ultimate compressive strength (**c**), and elongation (**d**) of PLA-Ti64 composite scaffolds (*p < 0.05, **p < 0.01, ***p < 0.001, ****p < 0.0001).

3D-printed scaffolds require acceptable mechanical properties to be used for BTE applications. Regarding the fact that the neat PLA with poor mechanical properties has failed to exhibit great potential in bio-based applications, tough Ti64 reinforcements were embedded through the matrix to fortify the mechanical properties of pure PLA. Compressive and tensile tests were performed to explore the effect of Ti64 on the mechanical properties of neat PLA. The obtained results are summarized in Table 2. As seen in Fig. 5a-d, when the filler concentration increased, the tensile modulus of the PLA matrix (1.3 GPa) was considerably magnified and reached the value of 2.0 GPa for PLA-9Ti64. The addition of filler was also found to be effective in improving the ultimate tensile strength and elongation of PLA, which were 31.4 MPa and 3.9, to the 35.1 MPa and 5.4 for PLA-3Ti64 sample. However, the upward trend for these parameters became reverse, and the mean values slightly decreased to 32.9 MPa and 4.1 for PLA-6Ti64 sample, followed by a significant fall to 30.5 MPa and 3.1 for PLA-9Ti64, which was approximately the same as that of obtained results in pure PLA. A similar pattern was also observed in compression results, with a difference that the PLA-6Ti64 sample acquired the highest value of ultimate compressive strength (Table 3 and Fig. 6a–d). According to these results, these scaffolds could be used for craniofacial bone^60^ and trabecular bone^61^. The justifying reasons behind the observed strengthening in tensile and compressive results can be explained as follows. According to some papers, the weak bonding between the reinforcements and matrix is directly linked to poor mechanical properties^62^. As discussed in the DSC results, the downward shift in the value of *T*_c_ by increasing the Ti64 concentration is a valuable piece of evidence to declare that PLA found Ti64 particles as preferable sites to nucleate, probably demonstrating PLA and Ti64 tendency to contact^63^. It is important to note that this interaction between the matrix and the filler allows the extra load, which PLA was unable to withstand, to be transferred effectively into Ti64 particles, which strengthens the composite^64^. Another reason for this phenomenon may be related to the excellent dispersion of filler without emerging agglomerations and microvoids on the surface of PLA-3Ti64 and PLA-6Ti64 composites. However, the decline in the strength of the PLA-9Ti64 composite can be a consequence of some agglomerations and voids demonstrated by FESEM images (Fig. 2). In order to obtain a better understanding of the mechanical behavior of the studied materials, the fracture surfaces of samples were studied and compared.

**Table 2:** Summary of the mechanical properties obtained from tensile tests.

Material	Tensile modulus (GPa)	Tensile yield stress (MPa)	Ultimate tensile strength (MPa)	Elongation (%)
Pure PLA	1.3	20.7	31.4	3.9
PLA-3Ti64	1.8	28.4	35.1	5.4
PLA-6Ti64	2.1	28.5	32.9	4.1
PLA-9Ti64	2.0	26.1	30.5	3.1

**Table 3:** Summary of the mechanical properties obtained from compressive tests.

Material	Compressive module (GPa)	Compressive yield stress (MPa)	Ultimate compressive stress (MPa)
Pure PLA	1.4	21.5	29.5
PLA-3Ti64	1.9	36.5	49.9
PLA-6Ti64	2.1	38.7	63.2
PLA-9Ti64	2.2	26.1	43.4

As depicted in Fig. 5e–h, although the fracture surface of pure PLA was smooth, which exhibits that PLA experienced a brittle fracture, a remarkable number of dimples and plastically deformed areas were observed on the surface of composites, which are representative of ductile fracture^65,66^. This result is directly consistent with the improvement of ductility (elongation) in tensile tests. Furthermore, apart from two types of porosities, including gas pore (formed during the filament manufacturing step (Type *A*)) and triangular interlayer pore (created between various individual layers during the printing step (Type *B*)), there is another type of porosity created by the pulled-out of Ti64 particles during the tensile test (Type *C*)^65^. These pores indicate that, before Ti64 debonding, the loaded tensile force was effectively transferred from matrix to fillers, thereby improving the mechanical properties of the PLA^67^.

As a result, even though the temperature of thermal degradation of composites was slightly elevated when the Ti64 concentration was increased up to 6 wt%, mechanical properties and stiffness of these composites were greatly enhanced. However, addition of 9 wt% Ti64 to PLA failed to improve mechanical properties, nor did it enhance thermal degradation. Therefore, the optimal interval of Ti64 was tuned between 3 and 6 wt%.

### Plasma treatment of scaffolds

**Figure 7:**
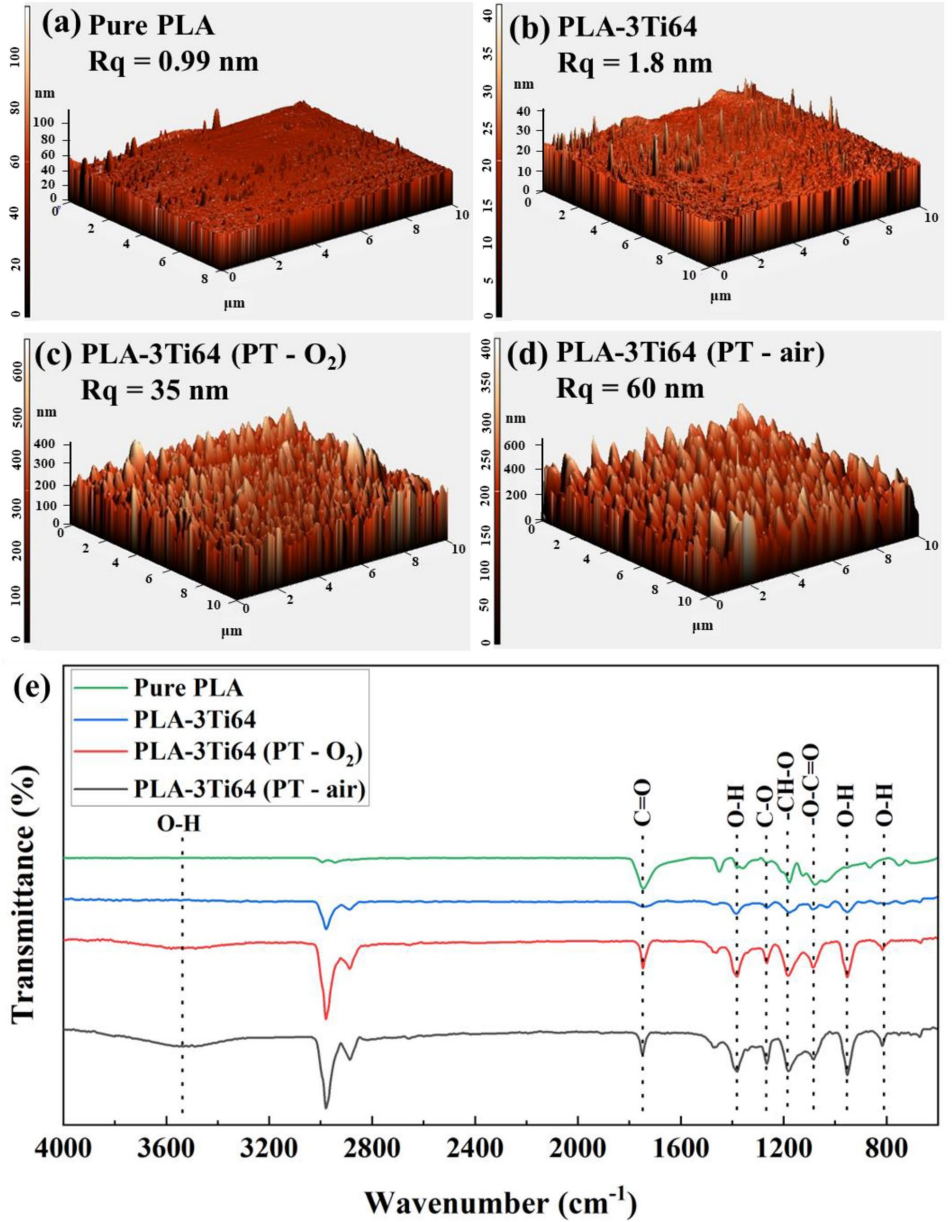
AFM results of 3D-printed samples of untreated pure PLA (**a**), untreated PLA-3Ti64 (**b**), PLA-3Ti64 (PT-O_2_) (**c**), and PLA-3Ti64 (PT-air) (**d**). (Images (**a**–**d**) were generated by NOVA softwarehttps://www.ntmdt-si.com/products/afm-features/nova-px-control-program (NT-MDT, *version 1.0.26.1443*)). ATR-FTIR of untreated and treated samples (**e**).

According to multiple surveys, polymers' smooth and hydrophobic surface is reluctant to interact with different functional groups, acting as a deterrent against cell adhesion and proliferation^68^. To overcome this challenge, non-thermal plasma treatment (PT) was employed to modify the PLA-3Ti64 surface in the presence of air and O_2_. Based on the atomic force microscopy (AFM) results presented in Fig. 7a and b, the slight increase in the root mean square roughness of PLA-3Ti64 sample (*R*_q_ : 1.8 nm) compared to that of pure PLA (*R*_q_ : 0.99 nm) is probably related to the deposited small Ti64 particles on the surface in the studied window of 10 µm × 10 µm. After surface treatment of the PLA-3Ti64 sample by O_2_ (Fig. 7c), the surface became rougher, and *R*_q_ reached the value of 35 nm, which was about fifteenth-fold that of the nontreated sample. Surprisingly, PT in the air was found to be more effective, as the *R*_q_ was approximately doubled (60 nm), demonstrating the incredible power of air treatment in roughening the surface (Fig. 7d).

PT is capable of bombarding polymer surfaces with various species such as ions, atoms, and excited molecules, which etches and modifies the surface by rupturing the polymer chains and forming the repetitive peak-valley structures in the nanoscale^69^. Additionally, PT can have a significant impact on the hydrophobicity of PLA by altering its surface. As can be observed in Fig. S2a and b, there was a decrease in the contact angle of PLA sample from 90.4 to 76.8° with the addition of Ti64 particles, which may be related to the increase in the roughness of PLA-3 wt%Ti64 scaffold compared to pure PLA scaffold^70^. When the PLA-3Ti64 sample was treated via O_2_ and air environments, the contact angles considerably diminished and reached the values of 54° and 46°, respectively (Fig. S2c and d). This higher hydrophilicity of plasma-treated samples can be explained by the fourier transform infrared spectroscopy (ATR-FTIR) results. Figure 7e shows the ATR-FTIR spectrum for pure PLA, PLA-3Ti64, PLA-3Ti64 treated with O_2_, and PLA-3Ti64 treated with air. The peak at 1080 cm^−1^ is attributed to –O–C=O bonding. At 1181 cm^−1^, the observed peak is for stretching –CH–O bonding. The peak at 1265 cm^−1^ is from C–O bonding of the ester group in PLA^71^. Also, there is a sharp peak at 1745 cm^−1^, which is C=O. The main difference between the composite sample and pure PLA is that the peak at 1745 cm^−1^ slightly decreased when Ti64 was added. According to some previous works, when the intensity of a peak at 1745 cm^−1^ was decreased, a partial interaction is probably made between two compounds, PLA and Ti64^72–74^. When the samples were plasma treated with O_2_ and air, the intensity of the oxygen-containing bonds, including –O–C=O, –CH–O, C=O, and C–O, increased directly associated with the number of oxygen bonds. As ions, atoms, and excited molecules incident the surface of PLA, they etch the surface and cause rupturing of the polymer chains. These sites start to interact with air to create oxygen-containing compounds. Moreover, the appearance of absorption peaks between 3650 and 3300 cm^−1^, at 950 cm^−1^ and 832 cm^−1^, as well as another intensified peak at about 1370 cm^−1^ after treatment, can probably be attributed to OH stretching vibrations responsible for increasing the hydrophilicity of the samples^75–77^.

### Cell viability, spreading, and proliferation

#### MTT assay

**Figure 8:**
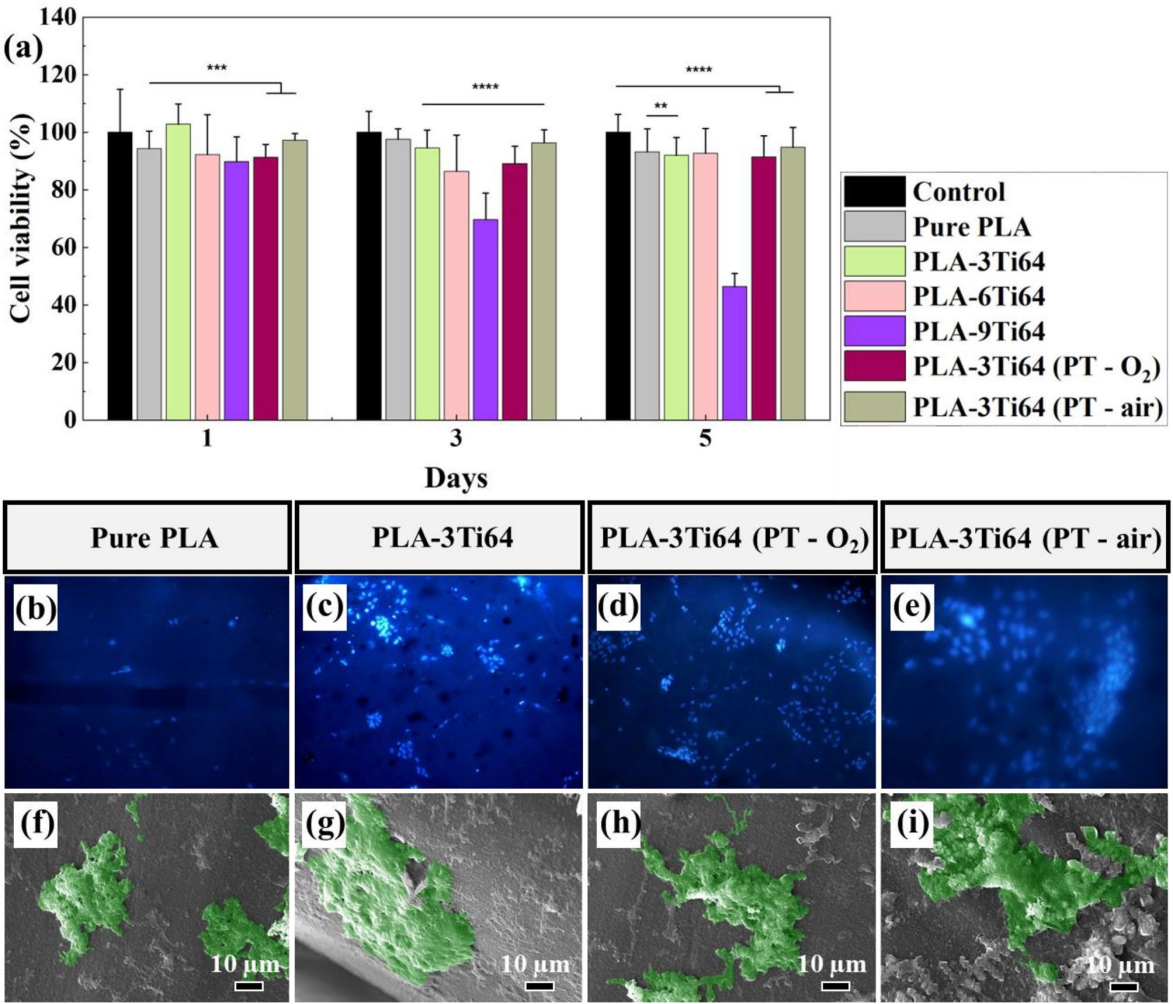
Cell viability and proliferation study. MTT assay analysis (**a**), DAPI staining (**b**–**e**), and SEM analysis (**f**–**i**) of fabricated specimens upon 5 days of cell culture. (*p < 0.05, **p < 0.01, ***p < 0.001, ****p < 0.0001).

The efficacy of incorporating Ti64 microparticles into PLA scaffolds was studied using a cell survival experiment. Figure 8a illustrates the cell viability analysis on days 1, 3, and 5 upon seeding WJ-MSCs on scaffolds, indicating that the developed PLA-based scaffolds possess acceptable cell viability compared with the control specimen.

The pure PLA specimen showed a similar trend on days 1, 3, and 5, compared to the control, while incorporating 3 and 9 wt% Ti64 affected the cell viability differently, resulting in the highest and lowest amount on day 1, respectively. The PLA-6Ti64 specimen possesses a cell viability value between PLA-3Ti64 and PLA-9Ti64. However, the obtained results indicate that plasma modification on the fabricated PLA-3Ti64 scaffolds resulted in lower cell viability on day 1 (91.281 and 97.268, with O_2_ and air, respectively) compared to the spare PLA-3Ti64 specimen.

On day 3, the maximum cell viability was dedicated to the pure PLA and plasma-treated specimens with air, with a statistically significant improvement (*p* < 0.0001). In contrast, the most harmful scaffold for cell viability was PLA-6Ti64. Furthermore, it was found that the plasma-treated specimens with air had the highest cell viability on day 5, with a significantly higher amount (*p* < 0.0001) than other specimens.

Based on the obtained results, it was evident that incorporating the Ti64 microparticles affects the cells' viability, and increasing their content had a more substantial impact as well. It could be justified as the ions and molecules that the additive microparticles released^78^; hence, depending on the utilized microparticles, the results would vary. Lee et al.^27^ reported that compared to cells attached to pure PLA scaffolds, those on PLA/Ti composite scaffolds were shown to be stretched and spread out more, and their proliferation was also boosted. Their work suggested that increasing the Ti content from 0 to 15 vol.% caused an enhancement in cell viability, proliferation, and osteogenic differentiation, compared to the pure PLA scaffolds.

Regarding the surface modification of prepared 3D printed scaffolds, it was shown that plasma treatment increased cell viability slightly, which was close parallel with previous studies. Roh et al.^43^ indicated that plasma treatment might alter the cells' behavior by altering the surface morphology and chemical features of the 3D scaffold. It seems that adjusting the scaffolds' surface and increasing the roughness result in an increase in the cells' attachment potential, affecting cell survival and spreading^79^.

Accordingly, the 6 and 9 wt% scaffolds were omitted from further cellular tests to perform applicable tests and develop augmented bone scaffolds. The evaluation was conducted considering four specimens, including Pure PLA, PLA-3Ti64, PLA-3Ti64 (PT-O_2_), and PLA-3Ti64 (PT-air).

#### DAPI staining and SEM analysis

4′,6-diamidino-2-phenylindole (DAPI), a convenient fluorescent stain, makes nuclear DNA detectable in both live and fixed cells^80^. Nuclei were identified, and overall cell morphology was evaluated using DAPI staining 21 days after cell seeding on the scaffolds (Fig. 8b–e). As these figures illustrate, the PLA-3Ti64 composite scaffolds showed a higher cell spreading and proliferation compared to the pure PLA scaffolds. Moreover, the results indicated that cell clustering in plasma-treated scaffolds made a higher cell spreading and proliferation, while the PLA-3Ti64 (PT-air) sample had elevated cell proliferation than plasma-treated specimens with O_2_. At the same time, both are superior compared to untreated composite and pure specimens. The obtained results correlate with previous findings, confirming that higher surface roughness results in higher cell attachment, spreading, proliferation, and functionalization^79^. Wang et al*.*^81^ reported that their attempts to adjust the 3D printed PLA-based scaffolds' surface via plasma treatment increased the hydrophilicity and nanoscale roughness, which boosted osteoblast differentiation and mesenchymal stromal cell adhesion and proliferation considerably. In this work, as discussed previously, the surface roughness of PLA-3Ti64 (PT-air), PLA-3Ti64 (PT-O_2_), PLA-3Ti64, and pure PLA were 60, 35, 1.8, and 0.99 nm, respectively. Given the data and the results, the cell attachment, spreading, proliferation, and morphology are considerable in plasma-treated samples, specifically the air plasma-treated specimen. Our findings on cell proliferation on PLA scaffolds show that the plasma surface treatment promotes cell proliferation. Nano-scaled surfaces generated by plasma treatment increased cell adhesion, proliferation, and even morphological alterations on the scaffold surface, as shown by the WJ-MSCs cell proliferation data. These findings were confirmed using SEM images from fixed cell-attached specimens (Fig. 8f–i).

### Osteogenic differentiation

#### Alkaline phosphatase (ALP) enzyme assay

**Figure 9:**
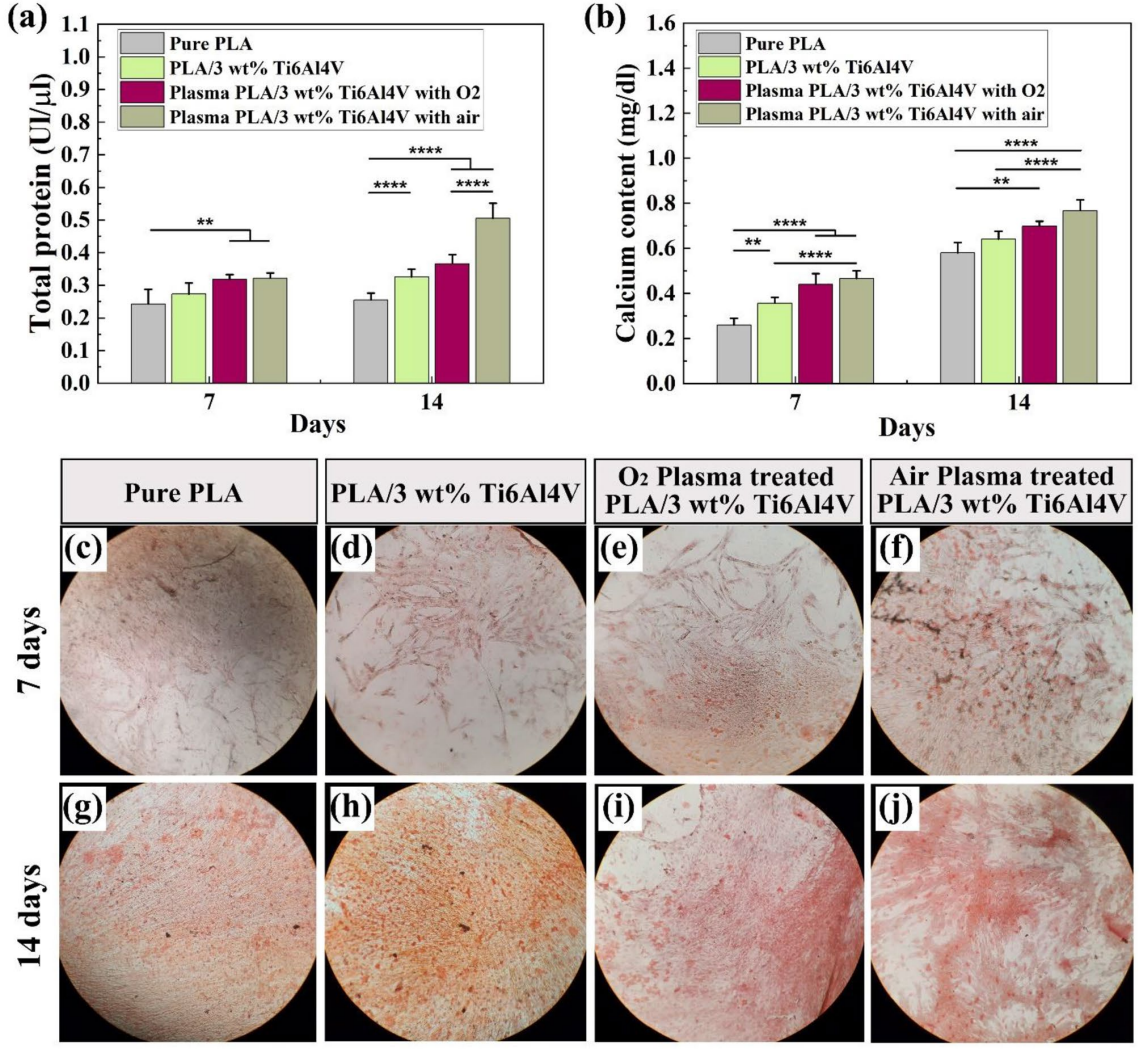
Osteogenic differentiation study. ALP activity analysis (**a**), ARS Staining analysis (**b**), and ARS staining at 7 days (**c**–**f**) and 14 days (**g**–**j**) upon cell culture. (*p < 0.05, **p < 0.01, ***p < 0.001, ****p < 0.0001).

To investigate the influence of plasma treatment on osteogenesis activity of WJ-MSCs seeded on 3D printed PLA/Ti64 composite scaffold, the ALP enzyme activity was measured, which corresponds to the early stage of osteogenic differentiation^82^. Figure 9a shows the ALP enzyme activity of WJ-MSCs seeded on the fabricated scaffolds for 7 and 14 days. Regarding the 7 days, it was found that incorporating 3 wt% Ti64 in PLA substrate increased the ALP enzyme activity, while this enhancement is not statistically significant. On the other hand, plasma surface treatment of the composite scaffolds elevated the ALP enzyme activity significantly, compared to the pure PLA specimens (*p* < 0.01), while the PLA-3Ti64 (PT-air) or PLA-3Ti64 (PT-O_2_) had no significant differences from each other. However, the situation was changed in 14 days upon cell culture on the developed 3D printed scaffolds. The PLA-3Ti64 scaffolds demonstrated a significantly elevated amount of ALP enzyme activity compared to the pure PLA specimens (*p* < 0.0001), whereas PLA-3Ti64 (PT-O_2_) had no significant improvement compared to the composite specimen. Interestingly, the plasma-treated specimens with air showed a considerable enhancement in ALP enzyme activity compared to the O_2_ plasma-treated and pure PLA specimens (*p* < 0.0001).

#### Alizarin red S (ARS) staining

Mature osteoblasts produce and deposit the mineral matrix, which mainly comprises calcium phosphate in the form of hydroxyapatite, as the ultimate stage of osteoblast differentiation. The capacity of WJ-MSCs to mineralize inorganic calcium in the middle of the differentiation phase, as measured by inorganic mineral deposition, indicates osteogenic differentiation. In this study, the ARS staining results illustrated the beneficial action of the plasma surface modified scaffolds on osteoblast extracellular matrix (ECM) mineralization. Figure 9b depicts the analysis diagram of ARS staining for WJ-MSCs seeded on prepared specimens for 7 and 14 days. It was observed that the average OD in 405 nm for pure PLA specimens was 0.2598 on day 7, while it raised to 0.3572 for PLA-3Ti64 substrate. Also, modifying the scaffolds' surface attributes by plasma treatment enhanced the deposited calcium content to 0.4402 and 0.4662 for O_2_ and air conditions, respectively. Figure 9c–f show the deposited calcium content from the differentiated cells on the prepared scaffolds for 7 days. The obtained qualitative results validate the numerical analyses, while the results for ARS staining positively correlate with the ALP assay.

The interesting point in this test is that, surface treatment of the specimens with plasma under O_2_ and air conditions increased the amount of calcium content to 0.6988 and 0.768, respectively, at 14 days; meaning that calcium deposition was significantly raised with roughing the surface and a better cell attachment, proliferation, activity, and differentiation, compared to the bare scaffolds (Fig. 9g–j).

It can be explained osteogenic scaffold potential by examining how plasma surface treatment alters the surface microenvironment. Since cell behavior has been shown to be influenced by the structure of the scaffold surface, the scaffold's chemical composition confers its biological capabilities^83^. In this research, as mentioned previously, it can be found that plasma treatment of the scaffolds enhanced surface roughness and altered the surface chemical composition (Fig. 7a–e). Surprisingly, the fact that increasing the surface roughness would enhance cell attachment, spreading, proliferation, and differentiation could be violated, depending on the material, scaffold, plasma treatment type, and cells used^84^.

## Conclusion

This study demonstrated successful 3D printing of PLA-Ti64 composite scaffolds with interconnected pores. The effects of adding different amounts (3, 6 and 9 wt%) of Ti64 to PLA on the mechanical and biological properties, as well as the effects of plasma treatment with oxygen and air on further enhancement of the biological properties of PLA-Ti64 scaffolds, in terms of cell attachment, proliferation, and differentiation, were investigated. The main conclusions can be summarized as the following.

1. The lack of detrimental defects, including micro cracks, delamination, and voids on the struts of the printed scaffolds, manifested the high quality of printed samples.
2. The addition of Ti64 to the PLA by 6 wt% significantly improved all mechanical properties, including compressive modulus, ultimate tensile strength, and ductility.
3. The glass transition temperature increased with increasing Ti64 content, indicating that Ti64 partly influenced their thermal properties. There was, however, a decrease in degradation, crystallization, and melting temperature.
4. The plasma treatment increased the hydrophilicity and roughness of the PLA-Ti64 scaffold surface. In this regard, plasma treatment with air was found to be more effective.
5. Addition of Ti64 particles in combination with plasma treatment resulted in improved adhesion, proliferation, and differentiation of Wharton's jelly mesenchymal stem cells.
6. The obtained results indicate that, among different studied samples, PLA-3Ti64 (PT-air) scaffolds showed better mechanical and bioactivity properties and met the ideal criteria for bone tissue engineering substitutes.

## Materials and methods

### Filament fabrication

Polylactic acid pellets (PLA 4032D, NatureWork LLC, USA) with an average molecular weight (Mw) of 19,600 kg/mol, spherical grade-23 Ti64 powder (AP&C, Canada) with a size distribution range of 10–50 microns (Fig. S3), and dichloromethane (DCM, biotech. grade 99.9%, Sigma-Aldrich, Germany) were used to prepare PLA/Ti64 composites. Details of the filament fabrication method is explained elsewhere^85^ and is described briefly here. Initially, PLA pellets were placed in an oven at 60 °C for 6 h to eradicate moisture. Then, Ti64 powder with three different concentrations (3, 6, and 9 wt%) was poured into a beaker containing 10 mL of DCM and stirred for 1 h to promote dispersion. After that, the resulting compounds were blended with dried PLA pellets by a hand mixer to evenly distribute the DCM/Ti64 mixture on the surface of the PLA pellets. The obtained PLA/Ti64 mixtures were placed in the oven at 70 °C for 16 h to remove DCM. Then, the final dried samples were added to a single-screw extruder equipped with two nozzles (temperature of 195 °C) and a screw speed of 35 rpm to prepare the final PLA/Ti64 filaments with a diameter of 1.75 mm. Two fans were installed at the outlet nozzle to descend the temperature of filaments. Finally, the coming filaments from the outlet nozzle were looped around a spool. Fig. S4 shows the picture of extruded pure PLA and PLA/6 wt% Ti64 filaments.

### Fabrication of PLA/Ti64 scaffolds through a 3D printing technique

In order to print scaffolds, a commercial material extrusion setup (Dayan K12S, Borna3D Inc., Iran) was used with a nozzle size of 400 µm. At first, scaffolds were designed by Solidwork software (version 23.0) in the cubic dimension of 1 × 1 × 0.8 cm with the strut width and pore size of 400 µm and 300 µm, respectively. Then, by the use of MankatiUM software (version 6.5), several printing parameters, including printing speed (35 mm/s), nozzle temperature (210 °C), bed temperature (55 °C), layer height (300 µm), and infill pattern (− 45°/ + 45°) were set. To reach the nominal porosity of 40%, the infill percentage of the samples was set at 60%. The printing process was followed by a pinch roller pushing the filaments to the heated nozzle. Then, the filament was melted and injected through the nozzle, with the melt depositing on a pre-heated print bed. Finally, the samples were detached from the bed and characterized by multiple tests.

### Plasma treatment (PT)

In order to improve surface roughness and promote cell attachment, oxygen and air plasma treatments were performed with a low-pressure plasma surface technology system (Zepto11, Diener, Germany) using a radio frequency (RF) of 13.56 MHz. The RF discharge power, O_2_ and air gas flow rates, and the working pressure RIE were 75 W, 15 sccm (standard cubic centimeter per minute), and 225 × 10^−3^ Torr, respectively. Both sides of 3D-printed scaffolds were treated for 6 min.

### Characterization of filaments and scaffolds

FESEM (Tescan Mira3, Czech Republic) equipped with EDX was used to evaluate the morphology and/or chemical composition of Ti64 powders, filaments, 3D-printed scaffolds, fracture surfaces, and also for cell attachment studies. The surface of samples was first coated with gold through sputter coating to make the surface conductive and prevent accumulation of electrical charge at the surface during SEM. A zoom stereomicroscope (SZ51, Olympus, Japan) was also used to assess the surface quality of the 3D-printed scaffolds. AFM (TS-150, NT-MDT, Russia) was used to investigate the topography and roughness of the plasma-treated and untreated 3D-printed scaffolds. AFM images were captured in the tapping mode at room temperature, and the root mean square (RMS) was calculated using NOVA software (version 1.0.26.1443).

TGA analysis (TA Instruments Q50, US) was conducted to study the thermal stability of bio-composite filaments. To achieve this, 10 mg of each sample was placed in an alumina crucible and heated at a 10 °C/min rate from 30 to 510 °C under an argon atmosphere. DSC analysis (TA Instruments Q100, US) was also performed to assess the crystallinity degree of the materials and their melting and crystallization behavior. In this regard, 10–15 mg of each sample was heated from 30 to 210 °C with a heating rate of 10 °C/min under an argon atmosphere. The degree of crystallinity (*X*_C_) was determined by the following Eq. ^86^:

1 $$Xc = \frac{{\Delta H_{m} - \Delta H_{c} }}{{\Delta H_{0} }}$$

where *ΔH*_m_, *ΔH*_c_, and *ΔH*_0_ are the enthalpies of melting, cold crystallization, and 100% crystalline PLA (93.1 J/g), respectively.

Shear viscosity measurement was investigated using a rotation Anton Paar MCR 301 rheometer with parallel geometries (plate diameter = 25 mm, gap = 1 mm) at 210 °C. The MFI test of filaments was carried out according to ASTM D1238 by using a Geotech GT-7100-MI apparatus. To this end, 2.16 kg of the load was applied to the plunger, and barrel temperature was maintained at 210 °C. Finally, material flown through the nozzle was observed for 10 min of testing.

The crystal structure of phases in 3D-printed scaffolds was examined using XRD analysis. XRD with filtered Cu–*K*α radiation (wavelength at 1.5405 Å) was carried out by a PW1730 Philips instrument at a current of 30 mA and voltage of 40 kV over the 2θ range of 5–80°.

The chemical functional groups of PLA/Ti64 scaffolds and their changes through plasma treatment were studied using FTIR (Thermo, US). The FTIR spectra were collected with a Perkin Elmer spectrometer in the range of 4000 cm^−1^ to 400 cm^−1^, using an attenuated total reflectance (ATR) modulus.

Contact angle measurement (WCA, Veho USB Microscope 400X, China) was employed to assess the hydrophilicity of various specimens. In this regard, 10 µL of distilled water was dropped on the proposed surface. The experiment was repeated for each sample, and the average results were reported.

Tensile and compression tests were carried out on 3D-printed specimens in accordance with ASTM D638 and D695 standards to determine the mechanical properties of PLA/Ti64 composites. A universal Santam (STM-20, Santam Co., Iran) testing machine was used to apply tensile and compression forces at constant crosshead speeds of 5 mm/min and 1.3 mm/min, respectively, at room temperature. Experiments were repeated at least three times to investigate the reproducibility of samples, and the mean values were reported.

### In vitro experiments

Wharton's jelly mesenchymal stem cells (WJ-MSCs) were purchased from SinaCell company (Iran) and subjected to an MTT test after being exposed to varying doses of PLA-Ti64 3D- printed scaffolds. To achieve this goal, 1 × 10^4^ cells were seeded onto 96-well culture plates and exposed to the prescribed scaffolds after a day of culture. After 1, 3, and 5 days, each well-received 100 mL MTT solution (0.5 mg/mL) was incubated for 3 h. The MTT solution was then withdrawn and replaced with 100 mL dimethyl sulfoxide (DMSO). Finally, the samples' absorbance (at 570 nm) was measured in triplicate using a microplate reader (BioTek Co., USA), and the findings for viability were calculated against the control sample and provided as a cell viability percentage.

For SEM analysis, cells were fixed on the surface of prepared specimens by immersing them in glutaraldehyde (2.5 wt%) for an hour and then rinsing them in phosphate buffered saline (PBS) solution. The dehydration was subsequently accomplished by immersing the sample at 37 °C for 5 min into each ethanol concentration (30, 50, 70, 80, 90, 95, and 99%). After receiving a gold coating, the samples were subjected to high-voltage SEM analysis.

To perform an ALP assay analysis, WJ-MSCs were cultured on the prepared scaffolds at a density of 2 × 10^4^ cells per well. The control and test groups refreshed the culture medium after 24 h. The media was taken on days 7 and 14, and the cells were washed twice with PBS, lysed in 200 L of 0.2 percent TritonX-100, and frozen/thawed three times at − 20/37 °C each. After centrifuging the cell lysates for 30 min at 3000 rpm, 50 L of the supernatant was transferred to a fresh 48-well plate and mixed with 200 L of *p*-nitrophenyl phosphate solution (Pars Azmoun Company, Iran) as directed by the manufacturer. The OD was measured at 405 nm using a microplate reader.

WJ-MSCs calcium deposition was detected by ARS staining. Approximately 4 × 10^4^ WJ-MSCs were seeded on the prepared scaffolds, each scaffold in each well of the 24-well plate, and the medium was refreshed after two days by the treatment groups. Cells were washed with PBS and fixed in 4% formaldehyde for 1 h after 7 and 14 days. After another wash, the cells were incubated for 40 min with 2 wt% Alizarin red (Sigma-Aldrich, USA). The cells were then washed four times with distilled water before being examined with an inverted microscope (Olympus, Japan).

In this work, an immunofluorescent evaluation was conducted to study and analyze the cell cycle and to detect cell nuclei utilizing DAPI staining. In order to investigate the occurrence of cell death and to detect whether cell death is of the apoptotic type or not, fluorescence microscopy (Leica, TCS SP-8, Germany) and DAPI staining (Sigma-Aldrich company, Germany) were used. In this staining, normal cells are seen as a uniform blue color. However, the nuclei of apoptotic cells are irregularly visible as bright blue dots due to chromatin condensation and fragmentation of the nucleus^87^. The DAPI solution was prepared by dissolving 1 mg of DAPI in 1 mL of distilled water. To evaluate cell death, seeded WJ-MSCs on the scaffolds were cultured for an hour before the cell surface was removed, washed with PBS, fixed with 4% paraformaldehyde, and stained with DAPI at 300 nm density.

### Statistical analysis

All graphs and statistical analyses were provided using Origin software (version 2021). *P*-values were calculated through one-way analysis of variation with Bonferroni multiple comparisons, where *P* < 0.05 was considered statistically significant. Each test was repeated at least three times, and results were reported as mean ± standard deviation (SD).

### Ethical approval and informed consent

No human or animals used in this study.

## Supplementary Information

Supplementary Figures.

## Data Availability

The datasets generated during and/or analyzed during the current study are available from the corresponding authors on reasonable request.
