# Supplementary material for: Enhanced bone tissue regeneration using a 3D-printed poly(lactic acid)/Ti6Al4V composite scaffold with plasma treatment modification
Source: Sci Rep. 2023 Feb 23;13:3139. doi: 10.1038/s41598-023-30300-z (PMC9950435; doi:10.1038/s41598-023-30300-z)
Supplement: Supplementary file 1 — Supplementary Figures. [file 41598_2023_30300_MOESM1_ESM.docx]

**Supplementary Information (SI)**

**Enhanced bone tissue regeneration using a 3D-printed Poly(lactic acid)/Ti6Al4V composite scaffold with plasma treatment modification**

Masoud Zarei^a,g*^, Motahareh Shabani Dargah^b,1^, Mahdi Hasanzadeh Azar^c,1^, Reza Alizadeh^a,*^, Fatameh Sadat Mahdavi^d^, Sayed.Shahab Sayedain^a^, Alireza Kaviani^e^, Mohammad Asadollahi^a^, Mahmoud Azami^f,g,h^, Nima Beheshtizadeh^f,g*^

*^a^ 3DBIOMETAL Research Group, Department of Materials Science and Engineering, Sharif University of Technology, Tehran, Iran.*

*^b^ Department of Biomedical Engineering, Amirkabir University of Technology, Tehran, Iran.*

*^c^ Department of Engineering Physics, McMaster University, Hamilton, Canada.*

*^d^ Department of Biotechnology Engineering, college of science, University of Tehran, Tehran, Iran.*

*^e^ Polymeric Materials Research Group (PMRG), Department of Materials Science and Engineering, Sharif University of Technology, Tehran, Iran.*

*^f^ Department of Tissue Engineering, School of Advanced Technologies in Medicine, Tehran University of Medical Sciences, Tehran, Iran.*

*^g^ Regenerative Medicine group (REMED), Universal Scientific Education and Research Network (USERN), Tehran, Iran.*

*^h^ Joint Reconstruction Research Center (JRRC), Tehran University of Medical Sciences, Tehran, Iran.*

Corresponding authors:

* Masoud Zarei

Email address: [m.zarei72@sharif.edu](mailto:m.zarei72@sharif.edu)

Address: Sharif University of Technology, Azadi Ave., Tehran, Iran.
Postal code: 11155-9466

* Reza Alizadeh

Email address: [r.alizadeh@sharif.edu](mailto:r.alizadeh@sharif.edu)

Address: Sharif University of Technology, Azadi Ave., Tehran, Iran.
Postal code: 11155-9466

* Nima Beheshtizadeh

Email address: [n-beheshtizadeh@razi.tums.ac.ir](mailto:n-beheshtizadeh@razi.tums.ac.ir)

Address: No. 88, Italia St, Qods Ave, Keshavarz Blvd. Tehran, Iran.

Postal Code: 14177-55469

**^1^** These authors contributed equally to this work.


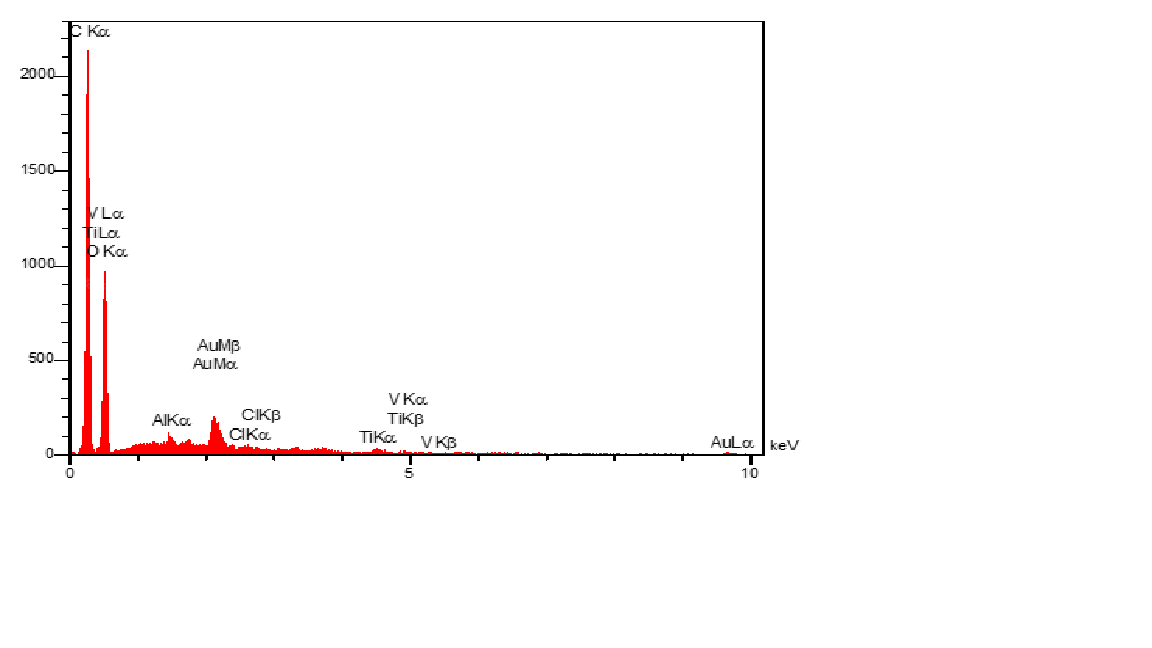


**Fig. S1.** EDX of PLA-Ti64 filaments.

**
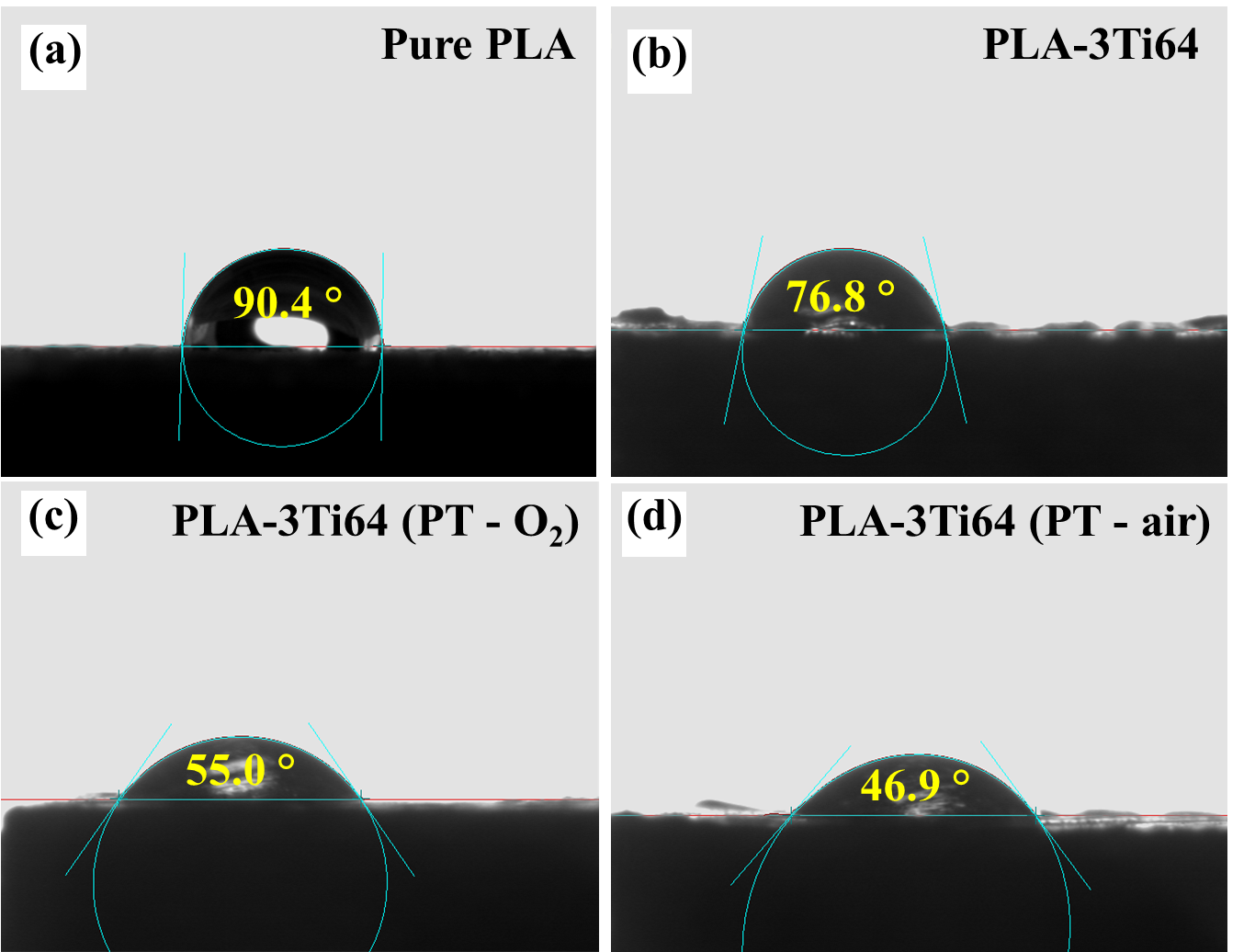
**

**Fig. S2.** Contact angle of pure PLA (a), PLA-3Ti64 (b), PLA-3Ti64 (PT-O_2_) (c), and PLA-3Ti64 (PT-air) (d) scaffolds.


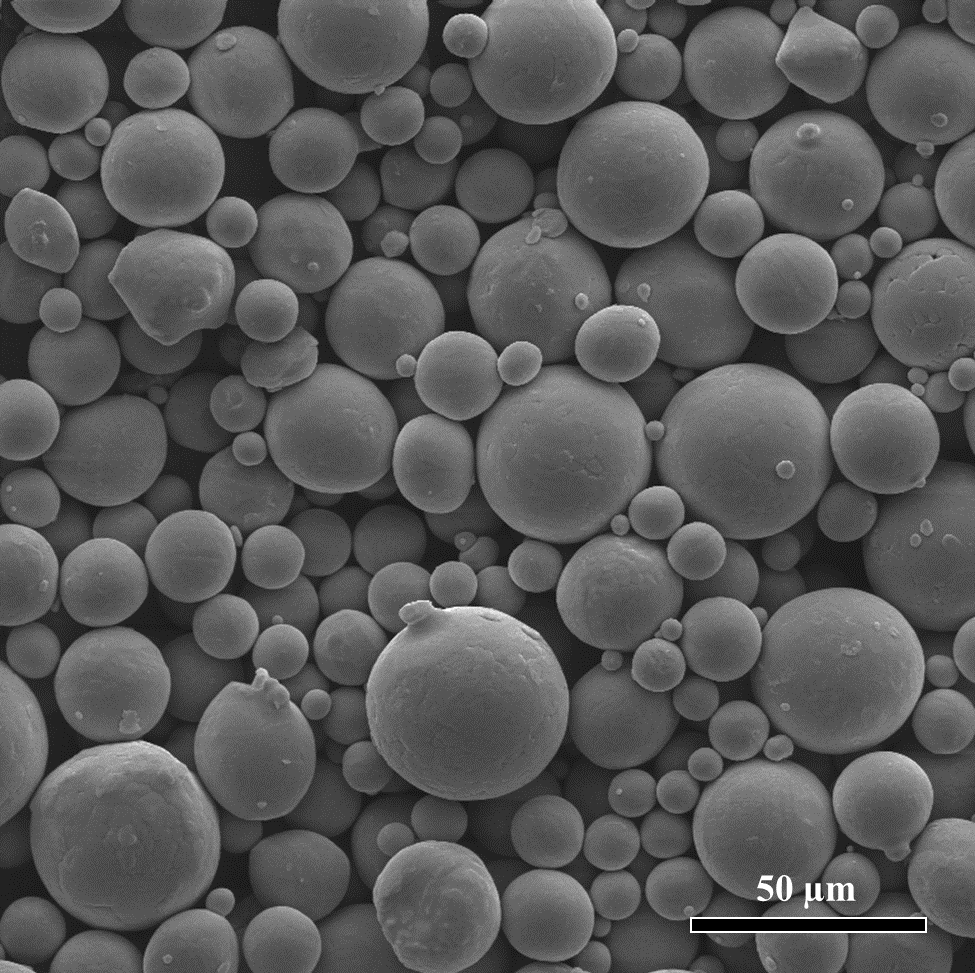


**Fig. S3**. FESEM of Ti64 powder.


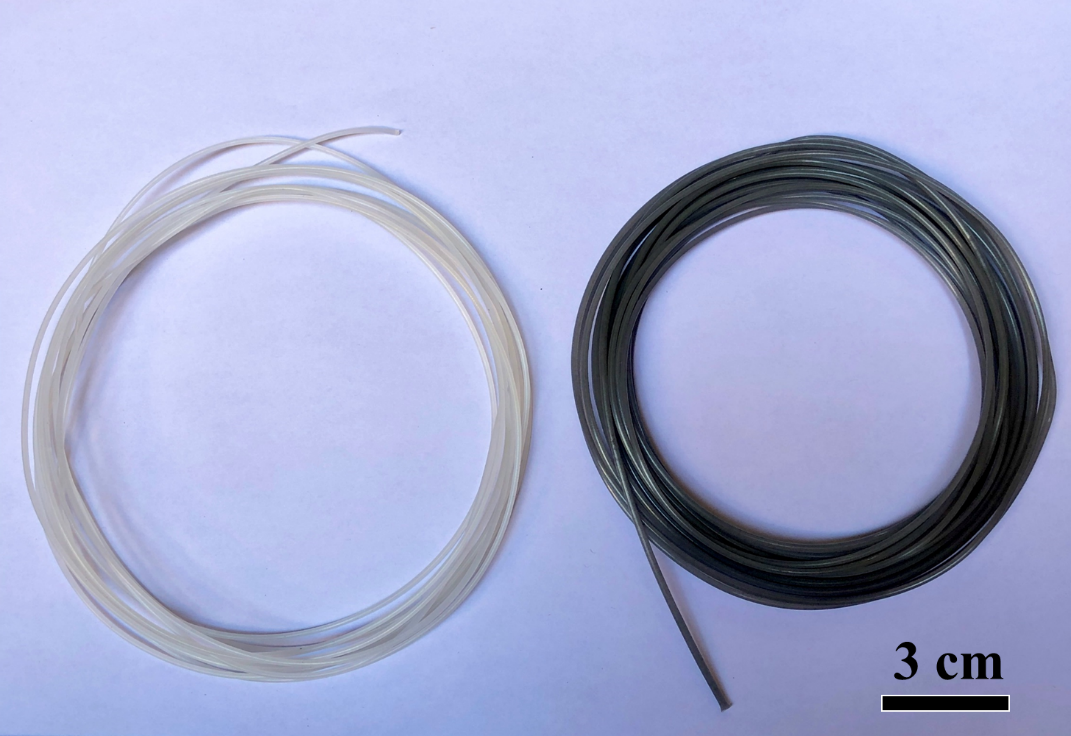


**(a)**

**(b)**

**Fig. S4**. Pure PLA (a) and PLA-6Ti64 (b) filaments.
